# Looking for a needle in a haystack – tackling rare diseases: an interview with Kym Boycott

**DOI:** 10.1242/dmm.020925

**Published:** 2015-05-01

## Abstract

Kym Boycott is currently a Clinical Geneticist at the Children's Hospital of Eastern Ontario (CHEO) and a Senior Scientist at the CHEO Research Institute, in Canada, where she tries to better understand mechanisms of rare genetic diseases and improve the management of pediatric patients with these conditions. Her interest in Medical Genetics dates back to her undergraduate studies at Queen's University in Kingston, when she was captured by Dr Patrick MacLeod's lectures on this subject. Thus, she embarked on a PhD in Medical Genetics and joined Dr Torben Bech-Hansen's lab at the University of Calgary, where she investigated the cause of a rare genetic form of vision loss. After completion of her PhD, she attended the medical school program at the University of Calgary and obtained her MD in 2005. Having both a PhD and MD allowed her to have a translational perspective from the beginning of her career. At CHEO, Kym and her group aim to bridge basic and clinical knowledge to quickly diagnose – by using next-generation sequencing – and improve the management of rare diseases, also known as orphan diseases. Kym is co-leader of the Canadian Rare Diseases Models and Mechanisms (RDMM) project, the goal of which is to connect basic scientists who work with animal models to clinician investigators studying rare diseases, thereby catalyzing investigation of disease mechanism and in some instances facilitating therapeutic configuration for rare diseases. In this interview, Kym shares with us her unique experience and expertise, fighting on the front line against rare diseases.

Kym Boycott was born in Australia. She obtained her Bachelor of Science with Honours degree in Biology at Queen's University in Kingston, Ontario, and received her PhD in Medical Genetics in 1997 from the University of Calgary, Canada. She then entered the medical school at the University of Calgary, graduating with her MD in 2000, and completed the Royal College of Physician and Surgeons training program in Medical Genetics in 2005. She is now working as a Clinical Geneticist at the Children's Hospital of Eastern Ontario (CHEO) and as a Senior Scientist at the CHEO Research Institute. She is also an Associate Professor in Pediatrics and holds a Tier II Research Chair from the Faculty of Medicine in Neurogenetics at the University of Ottawa. Kym's research aims to identify genes associated with rare diseases and to understand how dysregulation of gene products leads to the clinical presentation. She contributed to building the Canadian Rare Diseases Models and Mechanisms (RDMM) network, an invaluable collaborative platform bringing together experimental and clinical scientists to work on rare diseases, with the aim to speed up the discovery of causative pathways that could be targeted for therapeutic purposes.

**Figure:**
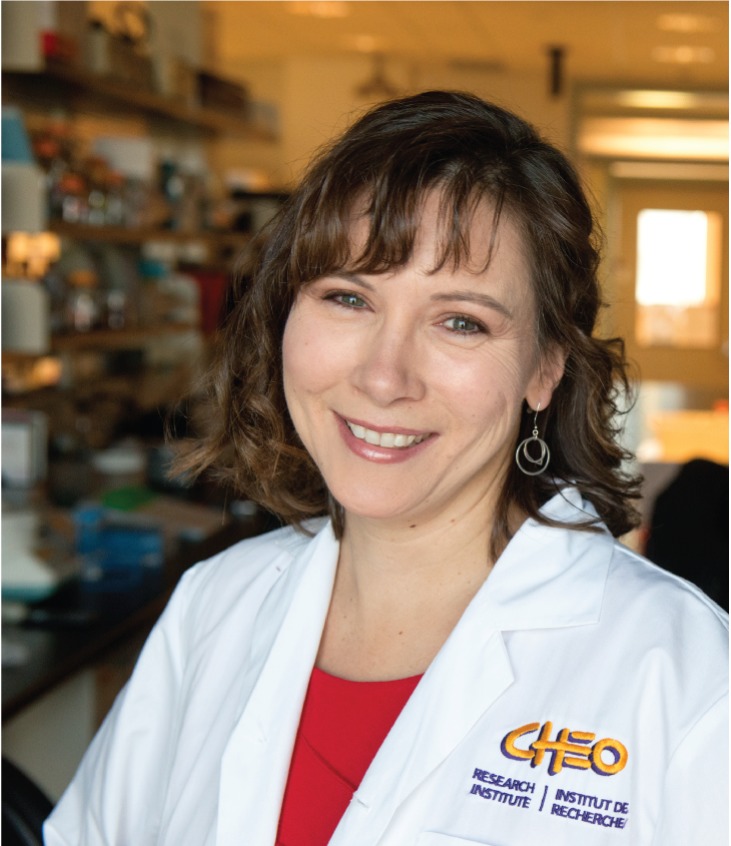

**You obtained your PhD in Medical Genetics and then completed the medical training program again in Medical Genetics. It seems that you were quite determined to study this discipline?**

Yes, I chose Medical Genetics early on. I would say I chose it in the second year of undergraduate studies at Queen's University, when Dr Patrick MacLeod came and gave a lecture on Medical Genetics to us in a biology class. Ever since then I loved genetics and I volunteered in his clinic, and completed my Honours project with him in the fourth year of my studies. So the intention when I left Queen's University was to continue studying genetics. I had gone to the University of Calgary to begin a Master of Science degree, but an important fork in the road appeared in my second year of graduate school. I went to my first eye-genetics meeting and I recognized that many of the attendees there were clinician scientists, working in both the clinic and their own research laboratories. They had the advantage of being able to see the patients with the diseases that they studied, and that translational perspective was attractive. I decided that this was the way I needed to go as well and I transferred to the PhD program with Dr Torben Bech-Hansen. When I finished graduate school, I went to the medical school at the University of Calgary while I continued working as a post-doctoral fellow in the same research laboratory; this kept me very grounded in research from the beginning.

“I went to my first eye-genetics meeting and I recognized that many of the attendees there were clinician scientists, working in both the clinic and their own research laboratories… I decided that this was the way I needed to go as well”

**How did the move to CHEO come about?**

When I finished the 5-year residency in Medical Genetics, I moved to Ottawa because there was a position available here that combined three critical factors: a great team in the clinic, opportunities at the CHEO Research Institute, and being located closer to my parents and sisters.

**Bringing together clinicians and lab researchers seems to be a priority for CHEO. Why is that so important for studying rare diseases?**

I think that's because for rare diseases there is so much that is unknown. Almost every patient that comes to see us in the clinic is potentially a research patient. If you look at all pediatric patients with a rare disease assessed in our clinic, at most we probably diagnose about 50% of these children. One of the most significant roles of a Clinical Geneticist in the care of these patients is to define what disease we are dealing with, so that we can be as informed as possible about the best management plan. So, if 50% of the time we are not able to make a diagnosis, it makes sense that many physicians practicing in clinical genetics also feel that clinical translational research is of upmost importance, and that patients who are without a diagnosis after many investigations – a diagnostic odyssey – should be eligible for research studies. You can imagine that clinical genetics in that sense really runs as a clinical service to some point and then transitions to a research service for those patients that are interested, and in my experience most families are indeed interested.

**What happens when clinical patients become research patients? How do you try to identify the genes associated with their rare diseases?**

One of the things we do in clinical genetics is to try to efficiently come to a diagnosis as quickly as possible to inform care for a particular child. We have to look at timeliness, cost effectiveness and the burden (in terms of investigations) of what we are about to ask families to undergo to reach the diagnosis. First, we evaluate what the best test or tests are for the child or adult with a very rare disease and, if these are unrevealing, some of these families are offered the opportunity to use next-generation sequencing of their coding genome to help identify a diagnosis. This is performed primarily in a research setting in Canada but is already available in some clinics in the USA and Europe. Generally, when we offer this possibility to a family, they accept it. They have come seeking an answer from our service and if we can't give it to them in the clinical setting, they are more than happy to participate in a research project that might give them the answers they are seeking. I co-lead a national research network called Care4Rare Canada that uses next-generation sequencing to try to identify the genes responsible for these rare diseases that we cannot diagnose in the clinical setting. We primarily use whole-exome sequencing that targets the coding portion of the genome and simultaneously analyses approximately 22,000 genes, about 1% of the genome. We have studied more than 500 families in this way and we are successful about 55% of the time, often identifying a previously unknown disease gene in the process. Because we are dealing with rare diseases, national and international collaboration is key to success, and we have many linkages, particularly through the International Rare Disease Research Consortium [www.irdirc.org].

“We primarily use whole-exome sequencing that targets the coding portion of the genome… We have studied more than 500 families in this way and we are successful about 55% of the time, often identifying a previously unknown disease gene in the process”

**So the benefit is having both clinicians and lab researchers working really closely together and being able to act quite quickly?**

It is patient-centered care, with research being an integrated part of care for patients with rare diseases. The team around them – including the clinicians, scientists and informaticians – they all work together to provide clarity for the family about what is happening to their child.

**You published an interesting paper in 2012 in which you proposed a generalizable preclinical research approach for orphan disease therapy that combines system-wide datasets with chemical informatics. Could you tell us the rationale behind it?**

Orphan diseases are so rare that the opportunity for big pharma to devote resources to them and to move them toward a novel therapy is probably not realistic for most diseases. What my colleague and research partner, Dr Alex MacKenzie, and I were trying to do in this paper is describe an approach by which one could use a high-throughput informatics pipeline to generate hypotheses about drugs currently approved for other disorders that might also be effective for a rare disease, the so-called ‘drug repurposing’. So we developed a list of approximately 100 rare diseases where we had sufficient insight into clinical course and mechanism, and Dr MacKenzie is tackling these conditions using this repurposing approach in his research program, with several promising leads identified so far. In our minds, repurposing is about trying to be more efficient and casting a wider disease net. We might find a possible treatment lead for only 1% or 2% or less than 5% of these rare diseases, but these therapies would come more quickly for those affected families because it is already a clinically approved medication. We study rare diseases based on the fact that we might be able to impact the disease course. It doesn't matter if there is just one patient or three patients or 50 patients, it's whether we can be successful; this is our primary outcome.

**You are a co-leader of the Canadian RDMM network. What is the scope of this network and where do model organisms fit in, concerning translational research?**

We are really good at finding previously unknown disease genes that cause rare diseases in Canada. This was recognized by the model organism community here and they advocated for an opportunity to become part of an organized translational pipeline – so we built one (alongside Dr Phil Hieter, University of British Columbia and Dr Janet Rossant, University of Toronto). The RDMM network gives us the opportunity to explore mechanistic insights for rare diseases of all sorts, as the expertise in Canada is significant and broad, and such insights can sometimes lead to ideas about therapeutic configuration.

At its core, RDMM is about making connections between clinician investigators and model organism scientists to study a particular rare disease together in a collaborative fashion. RDMM facilitates this work with start-up funds. We have captured information from the basic scientists in Canada that work with five animal models – yeast, fly, worm, zebrafish and mouse. There are about 350 such scientists who have registered in the RDMM database so far, representing more than 4000 rare-disease-related genes. When a clinician investigator enters the network it is because he or she has a gene of interest that is associated with a disease. It can be that either that gene has been associated with a given disease for a long time and there are therapeutic opportunities but nobody is studying this aspect, or it's a newly identified disease gene for which we have not yet got enough evidence to say it's definitively causative of a particular rare disease without more biological insight. The network database then helps us find suitable matches between clinician investigators and basic scientists. And when that happens, the basic scientist is awarded seed money of ∼25,000 Canadian dollars to do some initial experiments that would be expected to be completed quite rapidly, over about 6 months to a year. Ideally this work then continues with alternate funding and in that sense RDMM is seeding more labs to study rare-disease-relevant genes, which will ultimately benefit the patients [http://rare-diseases-catalyst-network.ca/].

“At its core, RDMM is about making connections between clinician investigators and model organism scientists to study a particular rare disease together in a collaborative fashion”

**There's always a lot of talk about how to bridge clinical and basic science, and this is a great example of how it works well. This looks like a great system to catalyze collaborations between basic and clinical research?**

Yes! The funding of RDMM will allow us to catalyze about 90 connections over 3 years. We have had 17 applications so far and have funding for 6 projects out the door.

**You are a clinician and a scientist, and you also hold a chair at the University of Ottawa. What is it like to have a foot in so many camps?**

Yes, I wear many hats that overlap. It can be tiring to keep pace – my team is always ten steps ahead of me! – but it's a really rewarding field of study.

**Since you've been working in clinical genetics for such a long time, where do you see this field in 10 years from now?**

I would really like to see much more integrated and translational care for patients with rare genetic diseases. I would like to see early diagnoses, insights into natural history, new patient management strategies, opportunities for clinical trials and overall a more significant impact on disease outcome.

**It seems like the field is moving towards this direction, but what else should be improved or developed?**

I think we need to further develop two sets of expertise. The first is being able to understand a patient's genomic information in the context of his or her health. I'm not talking about a population of healthy people and looking at their genome for things they might be at risk for when they're 75 years old. I'm talking about patients who have symptoms and signs that need to be investigated and managed. How do we understand the genomic information to really impact how we look after patients? We need to develop this skill set.

The second is how do we run effective small clinical trials in a network, such that we can show efficacy efficiently and thus make treatments much more readily available than they currently are now. Rare disease trials are hard to do, they're under-powered and underfunded, and so we have to find a strategy to address this challenge.

**How do you spend your time outside the hospital?**

I have two young, very busy kids. I spend a lot of time participating in their social activities and school work! I just like to be with them and my husband, whatever it is we're doing together – kids grow up so fast, so any time together is precious.

